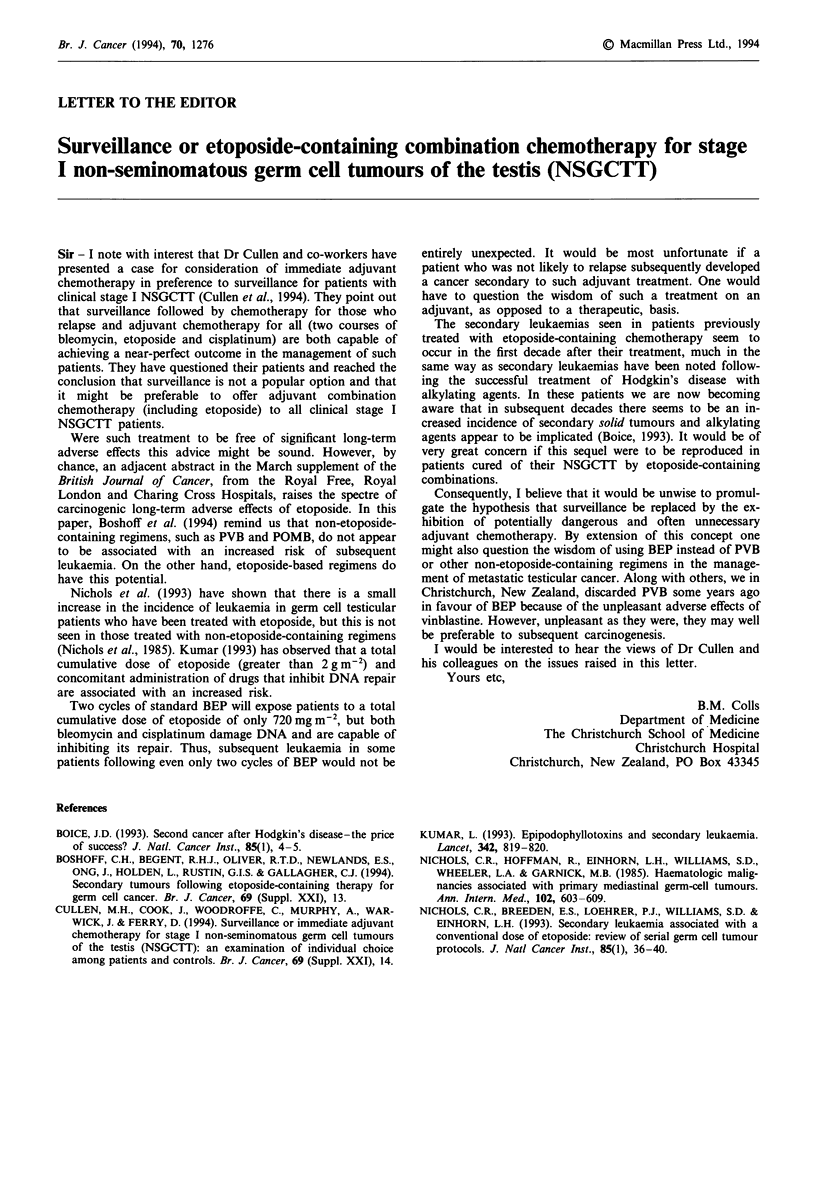# Surveillance or etoposide-containing combination chemotherapy for stage I non-seminomatous germ cell tumours of the testis (NSGCTT)

**DOI:** 10.1038/bjc.1994.487

**Published:** 1994-12

**Authors:** B. M. Colls


 © Macmillan Press Ltd., 1994

LETTER TO THE EDITOR

### Surveillance or etoposide-containing combination chemotherapy for stage I non-seminomatous germ cell tumours of the testis (NSGCTT)

**Sir** – I note with interest that Dr Cullen and co-workers have presented a case for consideration of immediate adjuvant chemotherapy in preference to surveillance for patients with clinical stage I NSGCTT (Cullen *et al.*, 1994). They point out that surveillance followed by chemotherapy for those who relapse and adjuvant chemotherapy for all (two courses of bleomycin, etoposide and cisplatinum) are both capable of achieving a near-perfect outcome in the management of such patients. They have questioned their patients and reached the conclusion that surveillance is not a popular option and that it might be preferable to offer adjuvant combination chemotherapy (including etoposide) to all clinical stage I NSGCTT patients.

Were such treatment to be free of significant long-term adverse effects this advice might be sound. However, by chance, an adjacent abstract in the March supplement of the *British Journal of Cancer*, from the Royal Free, Royal London and Charing Cross Hospitals, raises the spectre of carcinogenic long-term adverse effects of etoposide. In this paper, Boshoff *et al.* (1994) remind us that non-etoposide-containing regimens, such as PVB and POMB, do not appear to be associated with an increased risk of subsequent leukaemia. On the other hand, etoposide-based regimens do have this potential.

Nichols *et al.* (1993) have shown that there is a small increase in the incidence of leukaemia in germ cell testicular patients who have been treated with etoposide, but this is not seen in those treated with non-etoposide-containing regimens (Nichols *et al.*, 1985). Kumar (1993) has observed that a total cumulative dose of etoposide (greater than $2\,g\,m^{-2}$) and concomitant administration of drugs that inhibit DNA repair are associated with an increased risk.

Two cycles of standard BEP will expose patients to a total cumulative dose of etoposide of only $720\,mg\,m^{-2}$, but both bleomycin and cisplatinum damage DNA and are capable of inhibiting its repair. Thus, subsequent leukaemia in some patients following even only two cycles of BEP would not be entirely unexpected. It would be most unfortunate if a patient who was not likely to relapse subsequently developed a cancer secondary to such adjuvant treatment. One would have to question the wisdom of such a treatment on an adjuvant, as opposed to a therapeutic, basis.

The secondary leukaemias seen in patients previously treated with etoposide-containing chemotherapy seem to occur in the first decade after their treatment, much in the same way as secondary leukaemias have been noted following the successful treatment of Hodgkin's disease with alkylating agents. In these patients we are now becoming aware that in subsequent decades there seems to be an increased incidence of secondary *solid* tumours and alkylating agents appear to be implicated (Boice, 1993). It would be of very great concern if this sequel were to be reproduced in patients cured of their NSGCTT by etoposide-containing combinations.

Consequently, I believe that it would be unwise to promulgate the hypothesis that surveillance be replaced by the exhibition of potentially dangerous and often unnecessary adjuvant chemotherapy. By extension of this concept one might also question the wisdom of using BEP instead of PVB or other non-etoposide-containing regimens in the management of metastatic testicular cancer. Along with others, we in Christchurch, New Zealand, discarded PVB some years ago in favour of BEP because of the unpleasant adverse effects of vinblastine. However, unpleasant as they were, they may well be preferable to subsequent carcinogenesis.

I would be interested to hear the views of Dr Cullen and his colleagues on the issues raised in this letter.

Yours etc,

B.M. Colls
Department of Medicine
The Christchurch School of Medicine
Christchurch Hospital
Christchurch, New Zealand, PO Box 43345

### References

BOICE, J.D. (1993). Second cancer after Hodgkin's disease–the price of success? *J. Natl. Cancer Inst.*, **85**(1), 4–5.

BOSHOFF, C.H., BEGENT, R.H.J., OLIVER, R.T.D., NEWLANDS, E.S., ONG, J., HOLDEN, L., RUSTIN, G.I.S. & GALLAGHER, C.J. (1994). Secondary tumours following etoposide-containing therapy for germ cell cancer. *Br. J. Cancer*, **69** (Suppl. XXI), 13.

CULLEN, M.H., COOK, J., WOODROFFE, C., MURPHY, A., WARWICK, J. & FERRY, D. (1994). Surveillance or immediate adjuvant chemotherapy for stage I non-seminomatous germ cell tumours of the testis (NSGCTT): an examination of individual choice among patients and controls. *Br. J. Cancer*, **69** (Suppl. XXI), 14.

KUMAR, L. (1993). Epipodophyllotoxins and secondary leukaemia. *Lancet*, **342**, 819–820.

NICHOLS, C.R., HOFFMAN, R., EINHORN, L.H., WILLIAMS, S.D., WHEELER, L.A. & GARNICK, M.B. (1985). Haematologic malignancies associated with primary mediastinal germ-cell tumours. *Ann. Intern. Med.*, **102**, 603–609.

NICHOLS, C.R., BREEDEN, E.S., LOEHRER, P.J., WILLIAMS, S.D. & EINHORN, L.H. (1993). Secondary leukaemia associated with a conventional dose of etoposide: review of serial germ cell tumour protocols. *J. Natl Cancer Inst.*, **85**(1), 36–40.